# Revolving door plagiarism in medical research: breaking the vicious cycle

**DOI:** 10.1192/j.eurpsy.2025.2186

**Published:** 2025-08-26

**Authors:** R. K. Gupta, J. Yadav, R. Gupta

**Affiliations:** 1 Institute of Mental Health, Senior Professor and Director-cum-CEO; 2 Institute of Mental Health, Senior Resident, Rohtak; 3 Central Institute of Psychiatry, Junior Resident, Ranchi, India

## Abstract

**Introduction:**

The basic purpose of any research is to encourage and develop original thinking, critical analysis, and inculcate spontaneity and creativity. However, the online availability of a plethora of resources, sophisticated software, and artificial intelligence has opened floodgates to plagiarism, which is having adverse effect on developing minds for original research.

**Objectives:**

To comprehensively understand the current state of postgraduate research training, the challenges faced by students, and potential areas for improvement, particularly in fostering an environment conducive to original and unimpeded research.

**Methods:**

A cross-sectional web-based survey was conducted among medical students pursuing postgraduate courses in various specialties to address potential gaps in postgraduate research training. It comprised four sections with a total of twenty-seven items; Section one: Personal Data and Demographics; Section two: Exposure to Research Principles during Undergraduate Studies; Section three: Perception of postgraduate students towards Plagiarism Checks and Linguistic Barriers faced during dissertation writing; Section Four: An open-ended question inviting participants to suggest ways to promote the free flow of research ideas among young researchers. Survey data were exported to Microsoft Excel and analysed using descriptive statistics. Key themes and insights were derived from the suggestions provided in the fourth section of the questionnaire.

**Results:**

The survey questionnaire was completed by 130 respondents. The majority of the students were in their first or second year of postgraduate studies and represented various medical specialities. The majority of participants reported “low exposure” (n=86, 66%) to research methodology during undergraduate studies. 80% participants reported that they conducted a background research on the topic of their thesis and the most common challenge faced by them was lack of credible content in the online arenas. Almost one-third of the respondents reported that their original idea was blocked because of the “plagiarism checks”. Our survey participants represented linguistic diversity and 70.8% considered plagiarism as a marker of “linguistic proficiency”. 77.2% believed that conducting literature reviews exclusively in English, without considering texts available in other languages, hampers comprehensive research on a given topic.

**Conclusions:**

It is imperative to expose undergraduate students to “Practical aspects of research”, such as “Good research Practices”. Further, mentors should allow free flow of ideas, linguistic liberty, and encourage for in-depth discussion over these ideas, ensuring alignment with current scientific standards. An institutional level “Research integrity support” should be provided, including access to authentic plagiarism checking software and workshops focussing on research publication ethics.

**Disclosure of Interest:**

R. K. Gupta Consultant of: Nil, J. Yadav Employee of: Nil, R. Gupta Employee of: Nil

